# Demographic and Job-Related Predictors of Work-Related Quality of Life Among Healthcare Workers: Evidence from Public Sector Hospitals in Greece

**DOI:** 10.3390/healthcare13172142

**Published:** 2025-08-28

**Authors:** Olympia Christofilea, Maria Tsaousi, Loukia Psaridi, Panayota Sourtzi, Vasiliki Papanikolaou, George Dounias

**Affiliations:** 1 Department of Public Health Policy, University of West Attica, 11521 Athens, Greece; bpapanikolaou@uniwa.gr (V.P.);; 2 General Hospital of Korinthos, 20100 Korinthos, Greece; mtsaousi@hotmail.gr; 3 Red Cross General Hospital of Athens, 11526 Athens, Greece; 4 Department of Nursing, National and Kapodistrian University of Athens, 10679 Athens, Greece; psourtzi@nurs.uoa.gr

**Keywords:** public healthcare sector, WRQoL scale, occupational well-being, demographic predictors, psychosocial risk factors, job demands–resources model, cross-sectional study, economic crisis, COVID-19 pandemic

## Abstract

**Background:** Work-Related Quality of Life (WRQoL) is an essential aspect of the sustainability of the healthcare workforce, intimately connected to employee well-being, job fulfillment, and the standard of patient care. This research sought to evaluate WRQoL among healthcare employees in Greek public hospitals, concentrating on the influence of demographic and work-related factors in a healthcare system affected by prolonged economic and public health crises. **Methods:** A cross-sectional study was conducted with 1022 staff members from 23 hospitals in the 1st Health Region of Attica, utilizing the validated Work-Related Quality of Life Scale (WRQoL). Data were analyzed using non-parametric tests, including Chi-square and Linear-by-Linear Association analyses. **Results:** The findings showed that 44.3% of employees experienced low WRQoL, with the lowest ratings found among younger workers, those on temporary contracts, and individuals working in pediatric hospitals. Holding a leadership position, being over 40 years old, and having a permanent job were linked to notably greater well-being and job satisfaction. A significant portion of participants viewed the working conditions and autonomy as insufficient, particularly in demanding institutional environments. **Conclusions:** These results emphasize the necessity for focused policy measures to enhance working conditions, guarantee job stability, and reinforce organizational support structures. Tackling structural shortcomings in the healthcare system is crucial for protecting workforce stability and the standard of public health services.

## 1. Introduction

The idea of Work-Related Quality of Life (WRQoL) has received increased attention the last twenty years, particularly in fields like healthcare, where challenging work settings can greatly impact employees’ health, retention, and job performance. WRQoL includes various aspects of a person’s experience at work, such as mental health, satisfaction with their job, autonomy, work-life integration, environmental conditions, and stress levels [1].

Numerous theoretical models have been formulated to elucidate the processes by which work conditions influence the quality of life at work. Among these, the Job Demands–Resources (JD-R) framework has been extensively utilized in healthcare studies. This model suggests that job demands—like high workload, tight deadlines, and emotional stress—can result in strain and burnout if not counterbalanced by sufficient job resources, including autonomy, support, and acknowledgment [2,3,4]. Elevated job demands paired with inadequate resources not only worsen WRQoL but also obstruct employee engagement and motivation.

The Conservation of Resources (COR) theory provides an alternative perspective, suggesting that people aim to obtain and safeguard valuable resources (e.g., energy, time, support), and feel stress when these resources are endangered or lost [5]. During prolonged crises like economic austerity or pandemics, employees might experience ongoing resource depletion that gradually diminishes their well-being.

Additionally, the Self-Determination Theory (SDT) posits that employee motivation and well-being improve when three fundamental psychological needs are fulfilled at work: autonomy, competence, and relatedness. Work settings that encourage significant involvement, offer helpful feedback, and enhance peer support can boost intrinsic motivation, resilience, and, ultimately, the perceived quality of work life [6].

Furthermore, the Psychosocial Safety Climate (PSC) theory emphasizes that organizational practices emphasizing psychological well-being—like equitable workload distribution and transparent communication—can mitigate the adverse impacts of stress and foster a safer, healthier workplace [7].

The Greek healthcare system offers a distinct chance to analyze WRQoL in the context of extended stress. Between 2008 and 2018, the nation went through a major economic crisis that resulted in considerable cuts to public health funding, workforce reductions, and early retirements [8,9]. The subsequent COVID-19 pandemic (2020–2022) intensified pressure on the system, revealing long-standing shortages and heightening the emotional and physical strain on healthcare professionals [10,11].

Although international research has investigated WRQoL in different settings, limited attention has been given to healthcare professionals in Greece, particularly considering the combined effects of ongoing crises. Current studies often concentrate on individual professional groups (e.g., nurses or physicians) or on healthcare systems that are notably different from the Greek public model [12,13,14]. Additionally, while numerous studies mention theoretical frameworks such as JD-R and COR, only a limited number empirically implement them across various demographic and occupational groups.

This study seeks to fill these gaps by evaluating WRQoL in staff from 23 public hospitals within Greece’s largest health region. Employing the validated Work-Related Quality of Life (WRQoL) Scale [1,15,16], we investigate the relationship between important demographic (e.g., age, gender, education) and job-related (e.g., role, contract status, experience, hospital type) factors and workers’ views on WRQoL. Crucially, we assess how the legacy of the economic crisis and the impacts of the pandemic still affect work-life quality in the public healthcare sector.

Utilizing the theoretical principles of the JD-R, COR, and PSC models and implementing them on a substantial and varied sample, this research enhances both academic insight and policy formulation. It aims to guide specific workforce initiatives designed to enhance job satisfaction, lower stress levels, and bolster human resources within the Greek healthcare system.

Accordingly, the following research hypotheses were formulated:

H1: In the context of protracted socio-economic and health crises, health professionals perceive a deterioration in quality of life at work (WRQoL), as reflected in their subjective assessments of job satisfaction, well-being, and occupational stress.

MNBCVH2: An imbalance between job demands and available resources is associated with lower levels of work-related quality of life (WRQoL) among healthcare professionals, consistent with the theoretical propositions of the Job Demands–Resources (JD-R) model and the Conservation of Resources (COR) theory.

H3: Demographic (e.g., age, gender) and job-related (e.g., specialty, hospital type, experience) predictors are significantly associated with WRQoL dimensions.

## 2. Materials and Methods

This cross-sectional study was designed to assess the quality of working life among employees in Greek public hospitals. This study included a large population of health professionals (workers and managers from all specialties) employed in twenty-three hospitals of the largest health region in Greece and aimed, among other things, to find the determining factors that shape their quality of working life. Data collection took place from May 2023 to February 2024.

Sampling frame: Our study focused on the total number of staff (n = 23,941) in public hospitals (n = 23) in Attica’s 1st Health Region. To ensure a representative sample, cluster sampling was used. First, hospitals were divided into five categories based on their specific purpose and services provided: (a) general/main duty hospitals, (b) pediatric hospitals, (c) special purpose hospitals, (d) other/supportive hospitals, and (e) oncology hospitals. For better understanding of this classification, it is clarified that special purpose hospitals refer to facilities that provide highly specialized services within a specific medical field, such as dermatological, maternity-gynecological, ophthalmological, or rehabilitation care. Supportive hospitals, on the other hand, are small-scale facilities offering only basic healthcare services and typically operate as auxiliary units during the on-call rotation, addressing non-critical cases alongside main duty hospitals. For further details regarding the operational characteristics of each hospital category, see Table 1. Subsequently, the staff of the hospitals were divided into four clusters depending on their specialty: (a) medical, (b) nursing, (c) administrative/technical, and (d) other. Out of the total number of employees (23,941), 69% work in general/main duty hospitals, 13% in pediatric hospitals, 7% in special purpose hospitals, 3% in other/supportive hospitals, and 8% in oncology hospitals. Furthermore, 27% are physicians, 40% are nurses, 11% are administrative/technical staff, and 22% fall into other groups. To ensure that the sample (n) of each group is representative, the following parameters were set: n = 23,941 (total population); Z = standard deviation at 95% confidence level (Z = 1.96), D = tolerable difference (3%). Τhe sample size was calculated using Cochran’s formula, adjusted for finite populations. Given the foregoing, the total sample size (n) equates to 1022 employees. The proportion of staff per hospital type was maintained in the sample, as was the proportion of employees per cluster.

The selection of the 23 hospitals relied on their official categorization by the 1st Health Region of Attica, which designates institutional functions and range of services. All types of hospitals (general/main, pediatric, special purpose, supportive, and oncological) were incorporated to guarantee representative coverage of organizational varieties in the region. A minimum of one hospital from each category was chosen based on purposive criteria that guaranteed variation in size, specialization, and patient volume.

Inside each hospital, the human resources division provided comprehensive lists of qualified staff across all professional categories (medical, nursing, administrative/technical, other). A proportional stratified random sampling was conducted based on the size of each group to determine the number of participants per cluster, ensuring representation of the entire workforce and minimizing selection bias. Inclusion criteria required active employment during the data collection period, a minimum of six months of service in the same hospital, and voluntary participation. All eligible employees within each stratum were equally invited to take part, while response rates were monitored to verify comparability with the overall distribution of each staff group.

Participation was requested via formal internal notices released by hospital administrations, with further assistance from department supervisors to aid distribution. All participants gave informed consent and filled out the questionnaire anonymously and willingly, without any pressure or rewards. No hospital required compulsory participation.

Tools: The WRQoL scale, developed by researchers at the University of Portsmouth in the UK, was used for the purposes of the present study. It comprises six subscales that measure work control, general well-being, work–life balance, job satisfaction, occupational stress, and working conditions [1,15] and includes 24 items, each rated on a five-point Likert scale ranging from 1 = strongly disagree to 5 = strongly agree. The questionnaire has already been translated into Greek and validated for use in Greek-speaking populations [17].

Statistical analysis: Statistical analysis was conducted using SPSS version 26.0. Kolmogorov–Smirnov and Shapiro–Wilk tests were used to check the normality of data and both revealed deviation from normality for all dimensions of WRQoL. Hence, non-parametric techniques were utilized.

Descriptive measures (means, standard deviations, variances, frequencies, percentages) were used to describe the sample characteristics. Then, each of the WRQoL dimensions was categorized into three ordered levels (low, average, high) based on the percentile thresholds, as interpreted by the original authors of the questionnaire [18], and compared with each of the major demographic and job-related variables (e.g., age group, gender, hospital category) through the chi-square test. A *p*-value of <0.05 was considered indicative of statistical significance. The internal coherence check of the questionnaire was carried out with the Cronbach’s alpha indicator. We did not use multivariable statistical analysis methods because our data violated their assumptions.

To further establish associations, Pearson’s chi-square test was also used to determine the presence of statistically significant relationships while Linear-by-Linear Association (LBL) test was used to establish the direction and intensity of monotonic trends among ordinal variables. Having both of them applied not only served to establish significant associations, but also helped determine whether the associations existed in a systematic direction (e.g., advancing age was associated with improved WRQoL). This adds to the interpretive validity of the result.

Ethics: Ethical approval for the study was granted by the Research Ethics Committee of the University of West Attica (No. 37230/05-04-2023) and from all participating hospitals. This research was conducted in accordance with the principles of the Helsinki Declaration.

The sampling methods aimed to reduce selection bias and guarantee representativeness among various institutional types and job roles, thereby improving the generalizability of results within the Greek public hospital system.

## 3. Results

At the end of the survey period, 1022 questionnaires were collected (100% of the required number). The demographic and job-related characteristics of the participants are presented in Table 1. The mean age of the employees was 47.0 years (standard deviation = 10.4) and the median age was 48 years; the youngest participant was 21 years old and the oldest was 72 years old. The majority of the employees were women (71.3%). Regarding their professional characteristics, most employees were simple staff (79.5%), while 20.5% were managers. Additionally, 70.2% were permanent employees, while 29.8% were on contract. Most worked in main hospitals. The average total work experience was 20.4 years (standard deviation = 11.0), with the lowest value at 0.5 years and the highest value at 50 years. The main hospitals had a higher Bed Occupancy Rate (BOR), and the special ones followed.

Table 2 presents the Cronbach’s alpha coefficient for the six dimensions of the Work-Related Quality of Life (WRQoL) scale. The Cronbach’s alpha coefficient ranged from 0.71 to 0.86, indicating excellent reliability of the questionnaire.

### 3.1. Descriptive Results for the 6 Dimensions of the Work-Related Quality of Life (WRQoL) Scale and Classification Based on WRQoL Component Levels

#### 3.1.1. Descriptive Results

Table 3 shows the mean, standard deviation, and variance for each parameter of the quality of life in the sample. All dimensions of the questionnaire are at the moderate level of the scale. Therefore, the final score (74) also corresponds to the moderate level.

#### 3.1.2. WRQoL Component Levels

**Figure 1:**
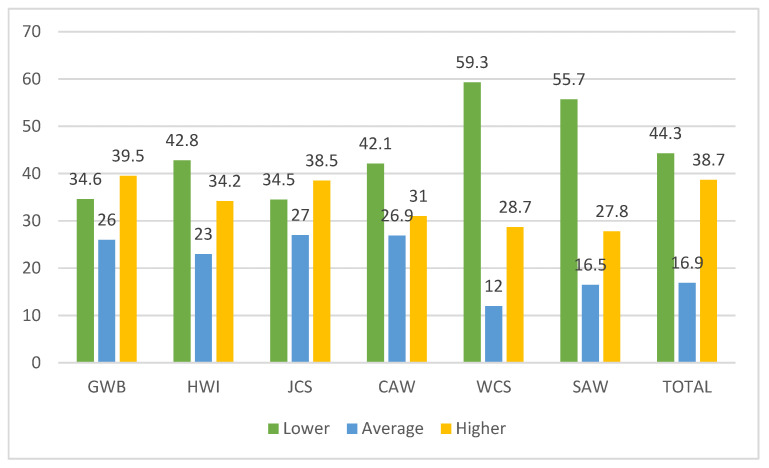
Level of quality of life of employees according to the Work-Related Quality of Life (WRQoL) questionnaire. WRQoL: Work-Related Quality of Life; GWB: general well-being; HWI: home–work interface; JCS: job and career satisfaction; CAW: control at work; WCS: working conditions; SAW: stress at work.

According to the Work-Related Quality of Life (WRQoL) scale, the level of quality of life is categorized as low, medium, or high. The low level of work-related quality of life based on the total score corresponds to 44.3% of employees, while 38.7% corresponds to a high level and 16.9% to the medium level. A total of 39.5% of respondents assess their general well-being as good, 57.2% rate their work-home interface as low or moderate, and 34.5% rate their job satisfaction as low. Additionally, 42.1% of respondents rank their ability to control work as low. The majority of employees, 59.3%, rate working conditions as low. Regarding stress at work, 27.8% report that it is high. The detailed score is reflected in Table 4 and Figure 1.

### 3.2. Relationship of Demographic and Job-Related Predictors to the Six Dimensions of WRQoL

Results of the relationship between demographic and job-related predictors and the six dimensions of the questionnaire (WRQoL), per dimension, are presented in Table A1, Table A2, Table A3, Table A4 and Table A5. It is noted that, in detail, the tables refer only to cases that are statistically significant.

#### 3.2.1. General Well-Being (GWB)

Employees’ perception of overall well-being differed notably based on age, job position, and employment type. Employees in senior roles, particularly those between 40 and 59 years old, consistently expressed greater levels of well-being. This pattern indicates that professional maturity and job security might alleviate work-related stress and enhance psychological resilience.

In contrast, younger employees—especially those below 40—indicated lower well-being levels, with the lowest ratings seen among physicians. This difference might indicate greater emotional requirements, reduced job autonomy, and limited organizational support at the start of one’s career. Employees on contract exhibited lower well-being than their permanent colleagues, emphasizing the effects of job insecurity.

The perception of well-being was also affected by educational background. Although university graduates indicated a decreased sense of well-being compared to those from technical schools, this observation might be linked to unrealistic expectations or insufficient use of their skills. The hospital category was significant, as staff in supportive hospitals indicated greater well-being, probably owing to lighter workloads and simpler patient cases. The detailed results of the GWB dimension are shown in Table A1.

#### 3.2.2. Home–Work Interface (HWI)

Perceptions of work–life balance were closely related to age and professional experience. Younger workers once again indicated notably lower HWI scores, hinting at challenges in balancing work obligations with personal duties. These results were particularly evident among physicians in children’s hospitals, where emotional strain and erratic schedules dominate.

In contrast, administrative personnel and employees with more than 30 years of experience indicated elevated HWI scores. This might indicate more reliable processes and well-developed coping methods. A leadership role was linked to better balance, probably because of increased freedom in managing time and distributing tasks.

The type of hospital became a significant element. Employees in special and supportive hospitals indicated improved home–work integration compared to those in pediatric or primary hospitals. These findings could be linked to variations in patient severity, work shift arrangements, and organizational backing for adaptable scheduling. The detailed results of the HWI dimension are shown in Table A2.

#### 3.2.3. Job and Career Satisfaction (JCS)

Job satisfaction exhibited a distinct upward trend as age and experience increased. Workers with over 20 years of experience reported the highest levels of satisfaction, whereas individuals under 40 or with fewer than ten years of service indicated significantly lower scores. These trends indicate that career development and the formation of professional identity require time to establish within the Greek public healthcare environment.

The leadership position was a significant indicator of satisfaction: more than 50% of all managerial employees rated their job satisfaction as high. This could arise from increased power in decision-making, enhanced visibility, and more consistent career paths. Conversely, discontent was focused among younger employees in subordinate roles.

The category of hospital also influenced satisfaction. Staff in specialized and supportive hospitals indicated greater satisfaction compared to those in pediatric departments, probably due to variations in workload intensity, emotional challenges, and opportunities for professional growth. These results underscore the significance of the institutional environment in influencing satisfaction. A detailed presentation of the results is provided in Table A3.

#### 3.2.4. Control at Work (CAW)

Experienced professionals, leaders, and permanent staff had greater control over work tasks and decision-making. Workers between the ages of 40 and 59 indicated considerably greater control compared to those younger than 40, emphasizing a growth in perceived independence. The leadership role was the most significant predictor, as almost half of all leaders indicated a high level of control.

Temporary workers and individuals at the beginning of their careers reported the least amount of control. Limited control over scheduling, processes, and patient care probably contributes to this perception, especially in hierarchical hospital environments. The difference in autonomy indicates systemic inequalities that could affect involvement and effectiveness.

The context of the hospital was important as staff in specialized and supportive hospitals experienced more autonomy compared to those in pediatric and main hospitals. This could pertain to smaller teams, more consistent routines, and reduced bureaucratic complexity, allowing for increased personal initiative. The analytical results are presented in Table A4.

#### 3.2.5. Working Conditions (WCS)

Overall, working conditions received low ratings, with significant differences observed among job categories and institutions. Medical staff in adult and children’s hospitals expressed the least satisfaction regarding their work setting, mentioning heavy patient volumes, scarce resources, and insufficient administrative assistance.

In comparison, administrative and technical personnel—especially in supportive hospitals—indicated better working conditions. These facilities generally handle less severe cases and provide more organized workflows, leading to a safer and more manageable work environment. The best ratings were observed in supportive hospitals, where almost 70% of staff considered conditions to be good.

Work experience additionally shaped perceptions. Workers with 30–39 years of service experienced the most favorable working conditions, possibly because of their increased knowledge of institutional systems and a sense of job stability. In contrast, junior employees expressed greater dissatisfaction, perhaps because of unfulfilled expectations or encountering less nurturing work environments. Detailed results are presented in Table A5.

#### 3.2.6. Stress at Work (SAW)

Stress levels varied among staff groups, with the highest levels reported by administrative and technical personnel. Approximately 41% of this cohort reported high stress levels, in contrast to 24% of physicians and 23% of nursing staff. This suggests that non-clinical personnel, although not directly involved in patient care, may encounter significant workload challenges with minimal organizational assistance.

Educational achievement also affected stress perception. Workers who had only completed primary education experienced greater stress, whereas individuals with postgraduate qualifications reported lower stress levels. This might indicate both the availability of resources (such as coping strategies and job mobility) and the complexity of tasks.

The hospital category influenced fluctuations in stress levels. Specialized and supportive hospitals reported the highest rates of employees experiencing high stress, likely due to diminished staffing or shared responsibilities. These patterns indicate organizational flaws that require attention to prevent prolonged burnout and disconnection. Detailed results are presented in Table A5.

### 3.3. Summary of the Six Dimensions of the Questionnaire Based on Demographic and Job-Related Predictors Affecting the Quality of Work Life of Employees in Greek Public Hospitals

After presenting the results for each dimension of the questionnaire in detail, Table 5 provides a concise overview of the examined prognostic predictors and their relation, or lack thereof, to the quality of working life of employees. According to the data in Table 5 of the demographic characteristics, age was the most influential (in five dimensions) followed by the level of education (in four dimensions). Gender did not show any differentiation across any dimension of the questionnaire. Work-related characteristics showed high statistics, with the most significant being the hospital category, which affects all dimensions of the quality of working life. This is followed by the job and the total years of work (five dimensions) and then the employment relationship and the job (four dimensions).

## 4. Discussion

The findings of this study highlight significant challenges in the work-related quality of life (WRQoL) among staff employed in public hospitals in Greece. A substantial proportion of participants reported low WRQoL, with pronounced deficits observed in areas such as autonomy, job satisfaction, working conditions, and perceived stress levels. These outcomes reflect deeper structural deficiencies within the public healthcare system—many of which have been magnified by successive national crises. Chronic underfunding, organizational inflexibility, and persistent resource shortages underscore systemic vulnerabilities, which have been exacerbated by prolonged socio-economic and public health pressures. Collectively, these findings provide robust support for Hypothesis H1, affirming that such crises have had a detrimental impact on healthcare professionals’ perceived quality of life at work.

The Job Demands–Resources (JD-R) model offers a valuable analytical framework for interpreting these patterns. The notably high job demands in Greek hospitals—manifested through excessive workload, understaffing, and emotional strain—are insufficiently counterbalanced by job resources such as managerial support, recognition, and effective supervision. This imbalance contributes to an increased risk of burnout and reduced employee engagement [2,3,4]. Complementarily, Conservation of Resources (COR) theory provides a theoretical lens for understanding the cumulative effects of resource depletion, including emotional exhaustion and job insecurity, which erode psychological resilience and professional identity. According to COR theory, resource retention and adequacy in the workplace are influenced by demographic and professional factors such as age, work experience, employment stability, and professional maturity. These factors play a pivotal role in enhancing both resilience and job satisfaction [5].

These theoretical perspectives align closely with our empirical findings and help to explain the variance observed across different professional and demographic groups. Specifically, the high job demands—such as workload intensity and perceived lack of control—were negatively associated with WRQoL dimensions, validating the strain-driven mechanism proposed by the JD-R model. Concurrently, COR theory sheds light on how prolonged exposure to uncertainty and psychological stress leads to a depletion of essential resources (e.g., mental resilience, perceived professional competence). According to this theory, such resource loss may trigger further psychological distress, reflected in respondents’ low scores in domains such as job satisfaction, general well-being, and psychological engagement. Thus, the current study illustrates the combined explanatory power of the JD-R and COR frameworks in interpreting empirical results and reinforces the theoretical validity of Hypothesis H2.

Of particular significance, the study found that employees under the age of 40 and those with fewer than 10 years of experience consistently reported lower levels of well-being, job satisfaction, and autonomy. This pattern suggests that younger professionals—especially those with temporary contracts—face greater job insecurity and insufficient organizational support to effectively acclimate to hospital settings. Limited perceived control among younger employees may reflect both hierarchical rigidity and ineffective onboarding practices, particularly in high-stress specialties such as pediatrics. For instance, 33.4% of employees aged 40–59 reported high job control, compared to just 23.3% of those under 40. While the 10% difference is moderate, it suggests that age is a relevant factor influencing perceived autonomy.

Moreover, leadership status and employment permanency emerged as protective factors for WRQoL. Across all subscales, managers and permanent staff consistently reported higher WRQoL. This likely reflects their increased access to institutional resources and decision-making authority. The difference in job satisfaction between leaders and regular staff was particularly noteworthy: 53.1% of leaders reported high satisfaction compared to only 34.1% of non-leadership staff—a substantial 19-percentage-point disparity. These findings are consistent with international literature, which identifies autonomy and perceived control as crucial determinants of job satisfaction within healthcare settings [18,19,20].

The Home–Work Interface (HWI) was identified as a key challenge, particularly for younger professionals and those working in pediatric healthcare. This may be attributable to emotionally intense patient interactions, time pressures, and insufficient organizational support for work–life balance. The emotional toll associated with pediatric care and exposure to child trauma likely contributes to the reduced well-being reported by younger physicians, a trend mirrored in international research [21,22,23,24].

In terms of General Well-Being (GWB), a striking disparity emerged between doctors and nurses: 56.6% of doctors reported low GWB, compared to just 23% of nurses. This suggests a substantial influence of professional specialty on psychological well-being and highlights the greater stress experienced by physicians, especially in demanding clinical environments.

Work-related stress (SAW) was also significantly associated with educational background and occupational role. Administrative and technical staff reported the highest stress levels (40.9%), markedly higher than those reported by doctors (24.1%) and nurses (23.7%). This gap of over 15% underscores a substantial relationship between job category and perceived stress. It may indicate a hierarchical imbalance where administrative and technical staff bear considerable responsibility but have limited authority, resulting in heightened stress. While nurses reported lower stress levels than administrative personnel, their perceptions of the work environment and autonomy still indicated significant limitations.

Persistently low ratings in the Working Conditions (WCS) dimension highlight the impact of structural and organizational challenges, including staff shortages, outdated infrastructure, and limited resources. Hospital type significantly influenced these outcomes: only 21.9% of pediatric hospital employees reported favorable working conditions, compared to 50.9% in specialized hospitals and 69.7% in supportive institutions. These findings underscore the critical role of institutional context in shaping perceptions of the work environment. Such challenges—intensified during the COVID-19 pandemic—are widely recognized in the literature as contributing to emotional exhaustion and reduced care quality [10,25]. Notably, assessments of working conditions were consistent with data from a parallel survey examining the safety climate in Greek public hospitals [26].

Overall, the findings point to a systemic misalignment between job demands and available resources, consistent with the predictions of both the JD-R and COR theoretical models. Importantly, the results also reveal significant disparities based on staff role, hospital type, age, and career stage, offering strong support for Hypothesis H3. These differences suggest that one-size-fits-all policy approaches are unlikely to be effective. Instead, tailored interventions—especially those targeting younger and contract-based staff—are essential for building workforce resilience.

From a policy perspective, several actionable strategies emerge. These include expanding permanent employment opportunities, strengthening leadership and managerial support systems, and developing specialized psychosocial support services. Furthermore, investing in continuous professional development and enhancing autonomy—particularly for early- and mid-career professionals—can mitigate the adverse effects of institutional stressors. The issue of brain drain, which has greatly impacted the Greek healthcare system [27,28,29], needs to be tackled with strategies for long-term retention and incentives for reintegration.

In conclusion, this study underscores the complex interplay between demographic and occupational factors and WRQoL in Greece’s public healthcare system. While certain findings resonate with broader trends observed in other Southern European countries—such as Italy, Spain, and Portugal—where public healthcare systems have similarly been strained by austerity measures, the interpretation and application of these results must remain context-specific. Future research should explore the role of institutional responses, cultural dynamics, and staffing structures in shaping WRQoL across comparable healthcare systems in Southern Europe.

## 5. Conclusions

The present study provides valuable insight into WRQoL within Greek public hospitals and contributes to national policy dialogue. Although comparisons with other Southern European healthcare systems can be insightful, the results should be applied with care and not broadly generalized. One must take into account contextual variability, institutional differences, and socio-political structures prior to applying these findings to different environments. The research provides a strong empirical basis for enhancing workforce resilience, especially in situations experiencing systemic pressure.

### Limitations

This study presents several limitations that should be considered when interpreting the findings, specifically in relation to the sample, study design, and analytical approach.

Delays in Research Implementation: The execution of this research was notably delayed due to the necessity of obtaining formal approvals from two authorities: the scientific councils and hospital administrations. These procedural delays, in conjunction with the involvement of 23 hospitals across diverse geographical regions, introduced significant administrative and time-related challenges, thereby extending the overall duration of the study.

Sampling Bias: The research focused exclusively on public hospitals in Greece, which limits the generalizability of the findings to professionals in the private healthcare sector or primary healthcare settings. Nevertheless, it is important to highlight that the public health system, particularly hospital-based care, was significantly impacted by both the economic crisis (2008–2018) and the subsequent COVID-19 pandemic (2020–2022). As such, the study’s focus on public hospitals is particularly relevant to understanding the unique challenges faced by these institutions during this critical period.

Limitations of the Analytical Approach: The study aimed to provide an extensive overview of the quality of working life (WRQoL) among employees in Greek public hospitals. It represents the first study to employ such a large and diverse sample, encompassing all professional groups and organisational structures within the largest healthcare region in Greece. However, the analytical approach primarily employed non-parametric bivariate statistical methods, which may be considered a limitation when compared to more advanced techniques such as factor analysis or structural equation modeling.

Rationale for Non-Parametric Methods: Given the historical context of the study, conducted in the aftermath of a prolonged economic crisis and during the COVID-19 pandemic, the primary aim was to descriptively assess how individual, occupational, and macrostructural factors (e.g., social uncertainty, understaffing, and occupational stress) influenced employees’ perceived WRQoL. Non-parametric methods were deemed appropriate for this purpose, as they facilitate the identification of statistically significant correlations and monotonic trends, especially when variables do not follow a normal distribution.

Deviations from Normality: Tests for normality (Kolmogorov–Smirnov and Shapiro–Wilk) revealed significant deviations from a normal distribution across all WRQoL dimensions. In addition, many of the variables were categorical or ordinal, further justifying the use of non-parametric approaches, such as chi-squared tests (χ^2^) and Linear-by-Linear Association, for robust statistical analysis and interpretation.

Consideration of Advanced Statistical Methods: While more complex statistical techniques could have been employed, they were not necessary for the scope of this research. The WRQoL instrument used had already been validated and weighted for the Greek population. The study’s objective was not to re-establish its factor structure but to apply it to comparative subgroup analyses. Additionally, the research was grounded in established theoretical models—the Job Demands–Resources (JD-R) and Conservation of Resources (COR) frameworks—which postulate that mismatches between job demands and available resources can lead to declines in employee well-being. These relationships can be effectively explored using structured bivariate analyses. Finally, due to the study’s cross-sectional design and correlational analysis, causal inferences cannot be made; future longitudinal research using advanced statistical methods (e.g., regression or SEM) is recommended to explore potential causal mechanisms.

## Figures and Tables

**Table 1:** Demographic and job-related characteristics.

Demographic Characteristic	n (%)
Age, years (n = 1017)	
<40	248 (24.4%)
40–59	687 (67.3%)
≥60	85 (8.4%)
age ^a^	47 (10.4)
Gender (n = 1020)	
male	292 (28.7%)
female	728 (71.3%)
Educational background (n = 1022)	
Elementary	31 (3.0%)
High school	145 (14.2%)
Technical education	397 (38.8%)
University	449 (43.9%)
Master’s/PhD	468 (45.8%)
**Job-related Characteristic**	**n (%)**
Hospital category (n = 1022)	
Main (11) ALOS 5.7, BOR 70.62%	710 (69.5%)
Pediatric (3)	137 (13.4%)
ALOS 4.45, BOR 46.12%	
Special (4)	57 (5.6%)
ALOS 8.15, BOR 61.42%	
Other supportive (3)	33 (3.2%)
ALOS 6.91, BOR 32.04%	
Oncological (2)	85 (8.3%)
ALOS 4.46, BOR 50.60%	
Personal categories (n = 1022)	
Doctors	274 (26.8%)
Nurses	414 (40.5%)
Administrative/technical	115 (11.3%)
Others	219 (21.4%)
Job position (n = 1022)	
Workers	813 (79.5%)
Leaders	209 (20.5%)
Employment relationship (n = 1022)	
Permanent	717 (70.2%)
Contract	305 (29.8%)
Total working years (n = 1022)	
Total working years ^a^	20.4 (11.0)
<10	445 (44.2%)
10–19	196 (19.5%)
20–29	188 (18.4%)
30–39	164 (16.0%)
≥40	29 (2.6%)

^a^ Average/standard deviation; ALOS: Average Length οf Stay; BOR: Bed Occupancy Rate.

**Table 2:** Cronbach’s alpha coefficient for the six dimensions of the Work-Related Quality of Life (WRQoL) scale for work-related quality of life.

Dimensions	Cronbach’s Alpha
General well-being (GWB)	0.86
Home–work interface (HWI)	0.82
Job and career satisfaction (JCS)	0.81
Control at work (CAW)	0.79
Working conditions (WCS)	0.82
Stress at work (SAW)	0.71

**Table 3:** Descriptive results for the six dimensions of the Work-Related Quality of Life (WRQoL) scale for work-related quality of life.

		GWB	HWI	JCS	CAW	WCS	SAW	TOTAL
N	Valid	1022	1022	1022	1022	1022	1022	1022
Mean		18.86	9.73	20.80	9.80	9.44	4.58	73.22
Median		19.00	10.00	21.00	10.00	10.00	4.00	74.00
Std. Deviation		4.878	2.874	4.364	2.596	2.643	1.798	14.400
Variance		23.796	8.262	19.044	6.742	6.986	3.235	207.359
Lower		6–14	6–8	15–19	3–8	3–8	2–3	29–65
Average		15–20	9–11	20–22	9–11	9–11	4–5	66–77
Higher		21–30	12–15	23–30	12–15	12–15	6–10	78–100

WRQoL: Work-Related Quality of Life; GWB: general well-being; HWI: home–work interface; JCS: job and career satisfaction; CAW: control at work; WCS: working conditions; SAW: stress at work.

**Table 4:** The component levels of quality of life of employees according to the Work-Related Quality of Life (WRQoL) scale (n = 1022).

Dimensions	Lower	Average	Higher
	n (%)	n (%)	n (%)
GWB	354 (34.6)	265 (26.0)	403 (39.5)
HWI	437 (42.8)	235 (23.0)	350 (34.2)
JCS	353 (34.5)	276 (27.0)	393 (38.5)
CAW	430 (42.1)	275 (26.9)	317 (31.0)
WCS	606 (59.3)	123 (12.0)	293 (28.7)
SAW	569 (55.75)	169 (16.5)	284 (27.8)
TOTAL	453 (44.3)	173 (16.9)	396 (38.7)

**Table 5:** Summary of the six dimensions of the questionnaire based on demographic and job-related predictors.

Dimensions	Demographic Predictors			Job Related Predictors				
	Age	Gender	Educational Background	Job Position	Employment Relationship	Job Category	Total Working Years	Hospital Category
GWB	√		√	√	√	√	√	√
HWI	√		√	√	√	√	√	√
JCS	√			√			√	√
CAW	√			√	√		√	√
WCS	√		√	√		√	√	√
SAW			√		√	√		√
TOTAL	5	0	4	5	4	4	5	6

## Data Availability

The data collected through questionnaires are not publicly available due to ethical restrictions, but may be provided by the corresponding author upon reasonable request.

## Abbreviations

The following abbreviations are used in this manuscript:

WRQoL	Work-Related Quality of Life
GWB	General well-being
HWI	Home–work interface
JCS	Job and career satisfaction
CAW	Control at work
WCS	Working conditions
SAW	Stress at work
JD-R	Job Demands–Resources model
COR	Conservation of Resources Theory
SDT	In line with Self-Determination Theory
PSC	Psychosocial Safety Climate Theory
SWB	Well-Being Theory
LBL	Linear-by-Linear Association
ALOS	Average Length οf Stay
BOR	Bed Occupancy Rate

## Appendix A

**Table A1:** General Well-Being (GWB) based on demographic and job-related predictors.

Predictors		Lower (%)	Average (%)	Higher (%)	*p* Value	Pearson χ^2^	LBL
**(i)** **Demographic predictors**							
	Age, years						
	<40	45.80%	24.00%	30.20%	<0.001	23.862	14.047
	40–59	29.80%	27.10%	43.10%			
	≥60	36.00%	22.70%	41.30%			
	Educational background						
	Elementary	38.70%	19.40%	41.90%	<0.001	35.213	22.846
	High school	24.10%	26.90%	49.00%			
	Technical education	28.30%	26.30%	45.50%			
	University	43.40%	25.70%	30.90%	0.593		
	Master’s/PhD	36.10%	26.30%	37.60%			
**(ii)** **Job-related predictors**							
	Job position						
	Workers	35.50%	27.80%	36.70%	<0.001	14.025	7.215
	Leaders	31.10%	18.70%	50.20%			
	Employment relationship						
	Permanent	30.30%	71.70%	43.20%	<0.001	26.183	14.376
	Contract	44.60%	24.60%	30.80%			
	Job category						
	Doctors	56.60%	23.40%	20.10%	<0.001	100.371	32.651
	Nurses	23.00%	30.80%	46.20%			
	Administrative/technical	30.40%	17.40%	52.20%			
	Others	31.10%	24.70%	44.30%			
	Total years worked at the hospital						
	<10	43.40%	22.90%	33.70%	<0.001	47.515	25.722
	10–19	33.80%	25.10%	41.00%			
	20–29	27.10%	33.50%	39.40%			
	30–39	21.30%	23.20%	55.50%			
	≥40	27.60%	44.80%	27.60%			
	Hospital category						
	Main	36.00%	27.10%	37.00%	<0.001	28.613	3.700
	Pediatric	29.20%	32.80%	38.00%			
	Special	35.10%	7.00%	57.90%			
	Other supportive	30.30%	6.10%	63.60%			
	Oncological	32.90%	25.90%	41.20%			

**Table A2:** Home–Work Interface (HWI) based on demographic and job-related predictors.

Predictors		Lower (%)	Average (%)	Higher (%)	*p* Value	Pearson χ^2^	LBL
**(i)** **Demographic predictors**							
	Age, years						
	<40	46.20%	28.40%	25.40%	<0.001	40.879	16.235
	40–59	25.30%	36.80%	37.90%			
	≥60	37.30%	30.70%	32.00%			
	Educational background						
	Elementary	32.30%	22.60%	45.20%	0.05	8.346	6.693
	High school	26.90%	32.40%	40.70%			
	Technical education	28.50%	36.30%	35.30%			
	University	36.50%	33.10%	30.40%	0.191		
	Master’s/PhD	34.80%	31.20%	34.00%			
**(ii)** **Job-related predictors**							
	Job position						
	Workers	33.00%	35.00%	32.00%	0.015	8.346	6.693
	Leaders	27.30%	30.10%	42.60%			
	Employment relationship						
	Permanent	28.90%	35.60%	3.60%	0.009	9.515	6.881
	Contract	38.70%	30.50%	30.80%			
	Job category						
	Doctors	39.40%	35.80%	24.80%	<0.001	28.069	22.035
	Nurses	33.80%	32.10%	34.10%			
	Administrative/technical	20.00%	35.70%	44.30%			
	Others	24.70%	34.70%	40.60%			
	Total years worked at the hospital						
	<10	36.00%	32.60%	31.50%	<0.001	38.396	11.654
	10–19	35.20%	41.80%	23.00%			
	20–29	25.50%	28.70%	45.70%			
	30–39	22.00%	34.10%	43.90%			
	≥40	41.40%	37.90%	20.70%			
	Hospital category						
	Main	31.50%	35.40%	33.10%	<0.001	26.455	3.368
	Pediatric	45.30%	2.30%	28.50%			
	Special	14.00%	40.40%	45.60%			
	Other supportive	15.20%	39.40%	45.50%			
	Oncological	30.60%	29.40%	40.00%			

**Table A3:** Job and Career Satisfaction (JCS) based on demographic and job-related predictors.

Predictors		Lower (%)	Average (%)	Higher (%)	*p* Value	Pearson χ^2^	LBL
**(i)** **Demographic predictors**							
	Age, years						
	<40	45.80%	22.90%	31.30%	<0.001	22.299	8.66
	40–59	29.90%	29.20%	40.90%			
	≥60	38.70%	25.30%	36.00%			
**(ii)** **Job-related predictors**							
	Job position						
	Workers	38.10%	27.80%	34.10%	<0.001	28.798	28.243
	Leaders	22.00%	24.90%	53.10%			
	Total years worked at the hospital						
	<10	41.10%	24.30%	34.60%	<0.001	31.339	12.553
	10–19	36.70%	30.10%	33.20%			
	20–29	26.10%	29.30%	44.70%			
	30–39	22.60%	30.50%	47.00%			
	≥40	51.70%	20.70%	27.60%			
	Hospital category						
	Main	35.20%	28.90%	35.90%	<0.001	33.161	4.131
	Pediatric	43.10%	24.80%	32.10%			
	Special	17.50%	26.30%	56.10%			
	Other supportive	15.20%	12.10%	72.70%			
	Oncological	37.60%	23.50%	38.80%			

**Table A4:** Control At Work (CAW) based on demographic and Job-related predictors.

Predictors		Lower (%)	Average (%)	Higher (%)	*p* Value	Pearson χ^2^	LBL
**(i)** **Demographic predictors**							
	Age, years						
	<40	38.50%	38.20%	23.30%	<0.001	19.144	15.95
	40–59	25.80%	40.80%	33.40%			
	≥60	24.00%	40.00%	36.00%			
**(ii)** **Job-related predictors**							
	Job position						
	Workers	32.60%	40.00%	27.30%	<0.001	33.011	32.956
	Leaders	15.30%	40.20%	44.50%			
	Employment relationship						
	Permanent	25.10%	42.30%	32.50%	<0.001	18.126	12.71
	Contract	38.40%	34.80%	26.90%			
	Total years worked at the hospital						
	<10	40.30%	35.60%	24.10%	<0.001	43.531	30.603
	10–19	38.00%	35.60%	26.30%			
	20–29	21.50%	43.70%	34.80%			
	30–39	19.80%	43.60%	36.60%			
	≥40	22.20%	44.40%	33.30%			
	Hospital category						
	Main	28.80%	42.00%	29.20%	<0.001	35.042	6.328
	Pediatric	43.8%	31.40%	24.80%			
	Special	12.30%	4.90%	43.90%			
	Other supportive	15.20%	30.30%	54.50%			
	Oncological	24.70%	38.80%	36.50%			

**Table A5:** Working conditions (WCS) based on demographic and Job-related predictors.

Predictors		Lower (%)	Average (%)	Higher (%)	*p* Value	Pearson χ^2^	LBL
**(i)** **Demographic predictors**							
	Age, years						
	<40	45.50%	34.50%	20.00%	<0.001	20.466	13.62
	40–59	31.60%	36.50%	31.90%			
	≥60	34.70%	36.00%	29.30%			
	Educational background						
	Elementary	35.50%	25.80%	38.70%	0.018	15.272	10.462
	High school	29.70%	33.10%	37.20%			
	Technical education	34.10%	35.10%	30.80%			
	University	37.70%	38.30%	23.00%	0.005		
	Master’s/PhD	35.90%	40.60%	23.50%			
**(ii)** **Job-related predictors**							
	Job position						
	Workers	38.50%	34.60%	26.80%	<0.001	15.785	13.468
	Leaders	23.90%	41.10%	34.90%			
	Job category						
	Doctors	40.50%	42.00%	17.50%	<0.001	49.054	11.852
	Nurses	37.50%	32.70%	29.80%			
	Administrative/technical	21.70%	27.00%	51.30%			
	others	32.90%	39.30%	27.90%			
	Total years worked at the hospital						
	<10	43.50%	35.20%	21.30%	0.002	24.276	20.099
	10–19	38.00%	37.10%	24.90%			
	20–29	34.10%	36.90%	29.00%			
	30–39	26.70%	35.00%	38.80%			
	≥40	37.00%	33.30%	29.60%			
	Hospital category						
	Main	36.20%	38.80%	25.00%	<0.001	65.209	16.986
	Pediatric	48.90%	29.20%	21.90%			
	Special	12.30%	36.80%	50.90%			
	Other supportive	15.20%	15.20%	69.70%			
	Oncological	31.80%	30.60%	37.60%			
